# Efficacy and safety of tigecycline monotherapy vs. imipenem/cilastatin in Chinese patients with complicated intra-abdominal infections: a randomized controlled trial

**DOI:** 10.1186/1471-2334-10-217

**Published:** 2010-07-21

**Authors:** Zhangjing Chen, Jufang Wu, Yingyuan Zhang, Junming Wei, Xisheng Leng, Jianwei Bi, Rong Li, Lunan Yan, Zhiwei Quan, Xiaoping Chen, Yunsong Yu, Zhiyong Wu, Dawei Liu, Xiaochun Ma, Robert Maroko, Angel Cooper

**Affiliations:** 1 Huashan Hospital, Fudan University, Shanghai, China; 2 Beijing Hospital, Beijing, China; 3 Peking University People's Hospital, Beijing, China; 4 Shanghai Changhai Hospital, Shanghai, China; 5 China PLA General Hospital, Beijing, China; 6 West China Hospital, Sichuan University, Chengdu, China; 7 Shanghai Xinhua Hospital, Shanghai, China; 8 Tongji Hospital, Wuhan, China; 9 First Affiliated Hospital, College of Medicine, Zhejiang University, Hangzhou, Zhejiang, China; 10 Shanghai Renji Hospital, Shanghai, China; 11 Peking Union Medical College Hospital, Beijing, China; 12 No. 1 Affiliated Hospital, China Medical University, Shengyang, LiaoNing, China; 13 Pfizer Inc., Collegeville, PA, USA

## Abstract

**Background:**

Tigecycline, a first-in-class broad-spectrum glycylcycline antibiotic, has broad-spectrum in vitro activity against bacteria commonly encountered in complicated intra-abdominal infections (cIAIs), including aerobic and facultative Gram-positive and Gram-negative bacteria and anaerobic bacteria. In the current trial, tigecycline was evaluated for safety and efficacy vs. imipenem/cilastatin in hospitalized Chinese patients with cIAIs.

**Methods:**

In this phase 3, multicenter, open-label study, patients were randomly assigned to receive IV tigecycline or imipenem/cilastatin for ≤2 weeks. The primary efficacy endpoints were clinical response at the test-of-cure visit (12-37 days after therapy) for the microbiologic modified intent-to-treat and microbiologically evaluable populations. Because the study was not powered to demonstrate non-inferiority between tigecycline and imipenem/cilastatin, no formal statistical analysis was performed. Two-sided 95% confidence intervals (CIs) were calculated for the response rates in each treatment group and for differences between treatment groups for descriptive purposes.

**Results:**

One hundred ninety-nine patients received ≥1 dose of study drug and comprised the modified intent-to-treat population. In the microbiologically evaluable population, 86.5% (45 of 52) of tigecycline- and 97.9% (47 of 48) of imipenem/cilastatin-treated patients were cured at the test-of-cure assessment (12-37 days after therapy); in the microbiologic modified intent-to-treat population, cure rates were 81.7% (49 of 60) and 90.9% (50 of 55), respectively. The overall incidence of treatment-emergent adverse events was 80.4% for tigecycline vs. 53.9% after imipenem/cilastatin therapy (*P *< 0.001), primarily due to gastrointestinal-related events, especially nausea (21.6% vs. 3.9%; *P *< 0.001) and vomiting (12.4% vs. 2.0%; *P *= 0.005).

**Conclusions:**

Clinical cure rates for tigecycline were consistent with those found in global cIAI studies. The overall safety profile was also consistent with that observed in global studies of tigecycline for treatment of cIAI, as well as that observed in analyses of Chinese patients in those studies; no novel trends were observed.

**Trial Registration:**

ClinicalTrials.gov NCT00136201

## Background

The management of complicated intra-abdominal infections (cIAIs) remains a challenge to physicians because of their polymicrobial nature coupled with the high risk of sequelae and mortality in severely ill patients with these infections [1-3]. While most infections contain a mixture of aerobic and anaerobic bacteria with a preponderance of Enterobacteriaceae (e.g., *Escherichia coli*) [1,2], resistant and uncommon organisms (e.g., *Enterococcus, Staphylococcus, Enterobacter*, *Pseudomonas *and *Candida *spp.) are often isolated in patients with nosocomial infection or tertiary peritonitis [4].

Selection of empiric antimicrobial therapy must consider the likelihood of encountering isolates that possess multiple resistance factors (e.g., extended-spectrum beta-lactamases [ESBLs], vancomycin-resistant enterococci [VRE]) [1,2]. Recently published treatment guidelines recommend broad-spectrum monotherapy or combination regimens (e.g., carbapenem monotherapy, third- or fourth-generation cephalosporins or fluoroquinolones plus metronidazole) for high-risk patients with severe or postoperative nosocomial intra-abdominal infections wherein polymicrobial infections and/or resistant flora are more prevalent [1,2]. Notably, inappropriate antibiotic choices have been linked to delayed clinical resolution, longer hospital stay, and an increased risk of mortality [5,6]. While adjunctive antimicrobial therapy is vital to achieving desired outcomes, surgical intervention is essential in the management of patients with cIAIs.

Tigecycline, a first-in-class expanded broad-spectrum glycylcycline antibiotic approved for use in patients with cIAIs, overcomes the 2 major mechanisms of resistance to tetracycline (i.e., drug-specific efflux pump acquisition and ribosomal protection) [7,8]. Tigecycline has broad-spectrum in vitro activity against bacteria commonly encountered in cIAIs, including aerobic and facultative Gram-positive and Gram-negative bacteria and anaerobic bacteria [9-11]. Furthermore, tigecycline has in vitro activity against multidrug-resistant bacteria such as VRE, ESBL- and carbapenemase-producing enteric Gram-negative bacteria, and methicillin-resistant *S. aureus *(MRSA) [12-14]. Tigecycline also exhibits linear pharmacokinetics and has a large volume of distribution, suggesting extensive tissue penetration [15].

Two global phase 3 double-blind trials, which compared the efficacy of tigecycline and imipenem/cilastatin in hospitalized patients with cIAIs, have demonstrated that tigecycline is efficacious for this condition [16]. Imipenem/cilastatin was chosen as the comparative agent because it has a wide spectrum of activity, it is effective in the treatment of hospitalized patients with intra-abdominal infections, and is widely available and used in the treatment of cIAI. In the current trial, tigecycline monotherapy was evaluated for safety and efficacy vs. imipenem/cilastatin in hospitalized Chinese patients with cIAI as a supplement to the 2 double-blinded pivotal global studies in cIAI [16].

## Methods

### Study design and treatment regimens

This study was a phase 3, multicenter, open-label trial of hospitalized Chinese patients at least 18 years of age who were candidates for or had undergone a laparotomy, laparoscopy, or percutaneous drainage of an intra-abdominal abscess and had a known or suspected diagnosis of cIAI. Specific enrollment criteria are outlined in Table 1. Following approval of the study protocol by the institutional review board or ethical review committee at each participating center, each patient or his or her legal representative provided written informed consent prior to undergoing any study procedures.

**Table 1:** Enrollment criteria

Inclusion*	Exclusion^†^
Men and non-pregnant, non-lactating women ≥18 years of age who required a surgical procedure for a complicated intra-abdominal infection (cIAI) cIAI defined as the following: An intra-abdominal abscess (including liver and spleen) that developed in a postsurgical patient after receiving standard antibacterial therapy (i.e., at least 48 hours, but not more than 5 days of antibiotics); Appendicitis complicated by perforation and/or a periappendiceal abscess; Perforated diverticulitis complicated by abscess formation or fecal contamination; Complicated cholecystitis with evidence of perforation or empyema; perforation of a gastric or duodenal ulcer with symptoms exceeding 24 hours; Purulent peritonitis or peritonitis associated with fecal contamination; Perforation of the large or small intestine with abscess or fecal contamination, or traumatic bowel perforation with symptoms lasting at least 12 hours before an operation	Preoperative suspicion of a diagnosis of spontaneous bacterial peritonitis, simple cholecystitis, gangrenous cholecystitis without rupture, simple appendicitis, acute suppurative cholangitis, pancreatic abscess, or infected necrotizing pancreatitis; Acute Physiologic and Chronic Health Evaluation (APACHE) II score greater than 30; Surgical procedure requiring that fascia or deep muscular layers be left open or expectation of planned abdominal re-exploration either in or out of the operating room; Use of immunosuppressive therapy that would decrease the patient's ability to eradicate the infection, including use of high-dose corticosteroids (e.g., 40 mg or more of prednisone or an equivalent per day for more than 3 weeks before randomization) or known diagnosis of acquired immunodeficiency syndrome; Current intra-abdominal infection known to be caused by one or more bacterial isolates not susceptible to either of the study drugs (e.g., *P. aeruginosa*, *Proteus mirabilis*); Active or treated leukemia or systemic malignancy that requires chemotherapy, immunotherapy, radiation therapy, or antineoplastic therapy within the 3 months before enrollment, or any metastatic malignancy to the abdomen with life expectancy < 6 months; Presence of any uncontrolled central nervous system disease; Significant hepatic disease (i.e., aspartate aminotransferase [AST] or alanine aminotransferase [ALT] level > 10 times the upper limit of normal [ULN] or total bilirubin value > 3 times the ULN) or acute hepatic failure or acute decompensation of chronic hepatic failure; Significant renal disease (i.e., calculated creatinine clearance < 41 mL/min/1.73 m^2 ^after adequate hydration); Neutropenia with absolute neutrophil count < 1000 mm^3 ^(however, neutrophil counts as low as 500 cells/mm^3 ^permitted if secondary to the acute infectious process); Concomitant treatment with ganciclovir

*Patients had to satisfy the inclusion criteria to be considered eligible for study participation

^†^Patients were ineligible for study participation if they had one or more of the listed exclusions

Patients were randomly assigned using a computerized enrollment system in a 1:1 ratio to receive tigecycline (initial 100-mg dose given by intravenous [IV] infusion over a 30-minute period, followed by 50 mg IV every 12 hours) or IV imipenem/cilastatin (500 mg/500 mg every 6 hours or dose-adjusted based on weight and creatinine clearance). Patients were to receive study drug for up to 2 weeks, unless deemed a treatment failure after at least 4 doses of tigecycline or 8 doses of imipenem/cilastatin.

Baseline aerobic and anaerobic cultures from the primary intra-abdominal site of infection and two sets of blood cultures were obtained within 24 hours of the first dose of study drug.

### Clinical and microbiologic evaluations

At serial visits throughout the study, the clinical status of the patient's intra-abdominal infection was assessed based upon the presence or absence of the following signs and symptoms: fever; localized or diffuse abdominal wall rigidity or involuntary guarding; abdominal tenderness or pain; ileus or hypoactive bowel sounds; nausea or vomiting. The clinical response to study drug was determined by the investigator at the test-of-cure (TOC) visit (12-37 days after therapy) and categorized as cure, failure, or indeterminate.

Microbiologic response by patient was categorized at the TOC visit as eradication, persistence, superinfection (i.e., the emergence of a new isolate was documented at the site of infection with worsening signs and symptoms of infection), or indeterminate. The microbiologic response for each baseline isolate at the TOC visit was categorized as eradication, persistence, or indeterminate.

### Safety and tolerability assessments

All patients who received at least one dose of study drug were evaluated for safety. Safety was assessed from clinical observations and findings from serial electrocardiograms (ECGs), serum chemistry, hematology, coagulation, and urinalysis tests. Adverse events (AEs) were recorded throughout the study period, up to and including the TOC visit or 14 days after last dose of study medication (whichever was longer), and were subjectively rated by the investigator as to their severity and relationship to the study drug. Investigators also recorded whether the AE resulted in temporary or permanent discontinuation and whether any remedial action was taken. Serious AEs (SAEs; i.e., those that were life-threatening, led to prolongation of the existing hospitalization, caused persistent or significant disability or incapacity, led to cancer, or death) were also recorded.

### Statistical analysis

Three primary populations of patients were assessed for safety, clinical, and bacteriologic outcomes; the modified intent-to-treat (mITT), microbiologic modified ITT (m-mITT), and the microbiologically evaluable (ME) populations. ITT patients who received at least one dose of study drug were included in the mITT population. Patients in the mITT population who had clinical evidence of complicated intra-abdominal infection comprised the clinical-mITT (c-mITT) population. The m-mITT population consisted of patients in the c-mITT population who had ≥1 isolate identified at the baseline assessment. Clinically evaluable (CE) patients were c-mITT patients who met all inclusion/exclusion criteria; did not receive concomitant antibiotics after the baseline intra-abdominal culture was obtained through the test-of-cure visit; received no more than 1 dose of a prohibited antibacterial treatment after the baseline intra-abdominal culture was obtained but before the first dose of study drug; received at least 5 days of study drug and between 80% and 120% of planned doses; had a test-of-cure visit 12 to 37 days after the last dose of study drug. Those patients included in the microbiologically evaluable (ME) population were CE patients who had at least one identifiable baseline bacterial isolate(s) taken from the primary site of infection that was susceptible to both study drugs and who had a microbiologic response assigned, i.e., eradication, persistence, or superinfection, at the TOC visit.

This trial was designed to enroll 200 patients. By assuming an evaluability rate of 50%, this would allow for the evaluation of 100 ME patients. The expected percentage of patients with a favorable clinical response (cure) was 70% at the TOC assessment. With a sample size of 50 evaluable patients in a treatment group, if 35 patients showed a favorable clinical response, then the 2-sided exact 95% CI would equal 55.4, 82.1.

The primary efficacy endpoints were clinical response at the TOC visit for the m-mITT and ME populations. Microbiological response at the TOC visit by patient and isolate was performed as a secondary analysis. Because the study was not powered to demonstrate non-inferiority between tigecycline and imipenem/cilastatin, no formal statistical analysis was performed for the primary and secondary efficacy endpoints of this study. Two-sided 95% confidence intervals (CIs) were calculated for the response rates in each treatment group for descriptive purposes using the "exact" method of Clopper and Pearson. Two-sided 95% CIs for differences between groups were calculated based on the asymptotic method corrected for continuity, except for differences in subgroup analyses where the Wilson score method corrected for continuity was used. Secondary efficacy analyses included the determination of susceptibility to tigecycline (MIC_50_, MIC_90_) and the development of decreased susceptibility (at least a 4-fold increase in MIC from baseline). Susceptibility was analyzed by using the Fisher exact test.

A post-hoc Cochran-Mantel-Haenszel analysis (stratified by protocol) was performed on the 2 co-primary efficacy endpoints to evaluate equality across the current trial and the 2 global cIAI studies [16]. The Breslow-Day test was used to evaluate equality across the strata with a *P *value of < 0.05 indicating statistical significance from study to study with respect to clinical response.

The Global Biostatistics and Programming Department of Wyeth Research performed all statistical analyses.

## Results

**Figure 1:**
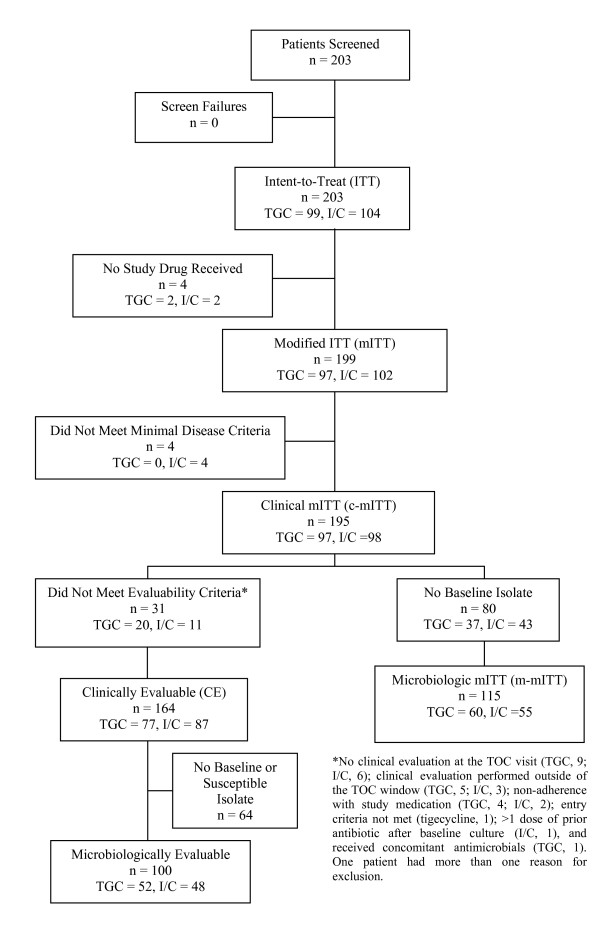
**Analysis populations and patient disposition: Tigecycline (TGC) vs. imipenem-cilastatin (I/C) in complicated intra-abdominal infections**.

The disposition of Chinese patients participating in this trial and the analysis populations are summarized in Figure 1. Overall, 199 patients received at least 1 dose of a study drug and comprised the mITT (safety) population.

The demographic and baseline medical characteristics for the mITT patients were comparable between the 2 treatment groups (Table 2) with the exception that tigecycline-treated patients were statistically significantly older (*P *= 0.021) and the severity of intra-abdominal illness was statistically significantly greater in the tigecycline cohort (mean APACHE II score was 5.1 for tigecycline vs. 4.1 for imipenem/cilastatin; *P *= 0.038). Complicated appendicitis was the most common intra-abdominal infection diagnosis in both groups (76.3% vs. 76.5% for tigecycline and imipenem/cilastatin, respectively).

**Table 2:** Demographic and baseline characteristics (mITT population)

	Tigecycline N = 97	Imipenem/cilastatin N = 102	*P *value*
**Mean ± SD age, years**	46.8 ± 18.2	41.0 ± 16.7	0.021
**Sex, n (%) male**	65 (67.0)	71 (69.6)	0.694
**Mean ± SD weight, kg**	63.2 ± 10.9	64.7 ± 11.1	0.335
**Mean ± SD creatinine clearance, mL/min**	100.9 ± 37.5	108.8 ± 37.1	0.138
**APACHE II score**			0.038
Mean ± SD	5.1 ± 3.9	4.1 ± 2.7	
Median (range)	4.0 (0.0 - 21.0)	4.0 (0.0 - 12.0)	
**Primary intra-abdominal diagnosis, n (%)**			0.509
Complicated appendicitis	74 (76.3)	78 (76.5)	
Complicated cholecystitis	5 (5.2)	7 (6.9)	
Perforated duodenal ulcer	3 (3.1)	7 (6.9)	
Peritonitis due to perforation of small intestine	6 (6.2)	2 (2.0)	
Peritonitis due to perforation of large intestine	4 (4.1)	2 (2.0)	
Perforated gastric ulcer	1 (1.0)	2 (2.0)	
Complicated cholangitis	2 (2.1)	0 (0)	
Post-traumatic peritonitis	1 (1.0)	1 (1.0)	
Liver abscess	1 (1.0)	2 (2.0)	
Perforated stomach	0 (0)	1 (1.0)	

NA = not available

*Categorical baseline demographic and medical variables were analyzed using the Fisher exact test. Continuous variables were compared using a one-way analysis of variance (ANOVA) model with treatment as a factor.

### Clinical efficacy

For the ME population, clinical cure rates were 86.5% for tigecycline and 97.9% for imipenem/cilastatin (95% CI for the difference, -23.5, 0.7) (Table 3). All patients had APACHE II scores ≤15. Corresponding clinical cure rates for the m-mITT population were 81.7% and 90.9%, respectively (95% CI for the difference, -23.4, 4.9). Clinical cure rates stratified by monomicrobial and polymicrobial infections are found in Table 3. For complicated appendicitis, by far the most frequent diagnosis in this study, clinical cure rates at the TOC visit for the ME population were 87.0% for tigecycline and 100.0% for imipenem/cilastatin (95% CI for the difference, -27.0, -0.6) (Table 4).

**Table 3:** Clinical cure rates* by analysis population at test-of-cure visit

	Tigecycline		Imipenem/cilastatin		Difference (Tigecycline-Imipenem/cilastatin)
**Population**	**N**	**% (95% CI)**	**N**	**% (95% CI)**	**% (95% CI)**

ME	45/52	86.5 (74.2, 94.4)	47/48	97.9 (88.9, 99.9)	-11.4 (-23.5, 0.7)
Monomicrobial	30/33	90.9 (75.7, 98.1)	25/26	96.2 (80.4, 99.9)	-5.2 (-22.0, 13.7)
Polymicrobial	15/19	78.9 (54.4, 93.9)	22/22	100.0 (84.6, 100.0)	-21.1 (-46.1, 2.2)
m-mITT	49/60	81.7 (69.6, 90.5)	50/55	90.9 (80.0, 97.0)	-9.2 (-23.4, 4.9)
Monomicrobial	32/38	84.2 (68.7, 94.0)	27/29	93.1 (77.2, 99.2)	-8.9 (-26.0, 10.7)
Polymicrobial	17/22	77.3 (54.6, 92.2)	23/26	88.5 (69.8, 97.6)	-11.2 (-35.8, 13.0)
CE	67/77	87.0 (77.4, 93.6)	83/87	95.4 (88.6, 98.7)	-8.4 (-18.3, 1.5)
c-mITT	78/97	80.4 (71.1, 87.8)	88/98	89.8 (82.0, 95.0)	-9.4 (-20.3, 1.6)

*Clinical responses defined as: *Cure*--the course of study drug and the initial intervention (operative and/or radiologically guided drainage procedure) resolved the intra-abdominal infectious process; *Failure*--the patient required additional antibacterial therapy other than the study drug, the patient required additional surgical or radiologic intervention to cure the infection, death due to infection occurred after 48 hours of therapy or a treatment-related adverse event (AE), or the patient received an extended course of study drug (i.e., > 120% of the planned number of doses); and *Indeterminate*--the patient was lost to follow-up, or died within 48 hours after the first dose of study drug for any reason, or died after 48 hours because of noninfectious-related reasons (as judged by the investigator).

**Table 4:** Clinical cure rates by diagnosis at test-of-cure visit (ME population)

	Tigecycline		Imipenem/cilastatin		Difference (Tigecycline-Imipenem/cilastatin)
**Diagnosis**	**N**	**% (95% CI)**	**N**	**% (95% CI)**	**% (95% CI)**

Complicated appendicitis	40/46	87.0 (73.7, 95.1)	45/45	100 (92.1, 100.0)	-13.0 (-27.0, -0.6)
Complicated cholecystitis	2/2	100.0 (15.8, 100.0)	0/0	NA	NA
Peritonitis	2/3	66.7 (9.4, 99.2)	2/3	66.7 (9.4, 99.2)	0.0 (-62.7, 62.7)
Gastric/duodenal perforation	1/1	100.0 (2.5, 100.0)	0/0	NA	NA

NA - not available

**Figure 2:**
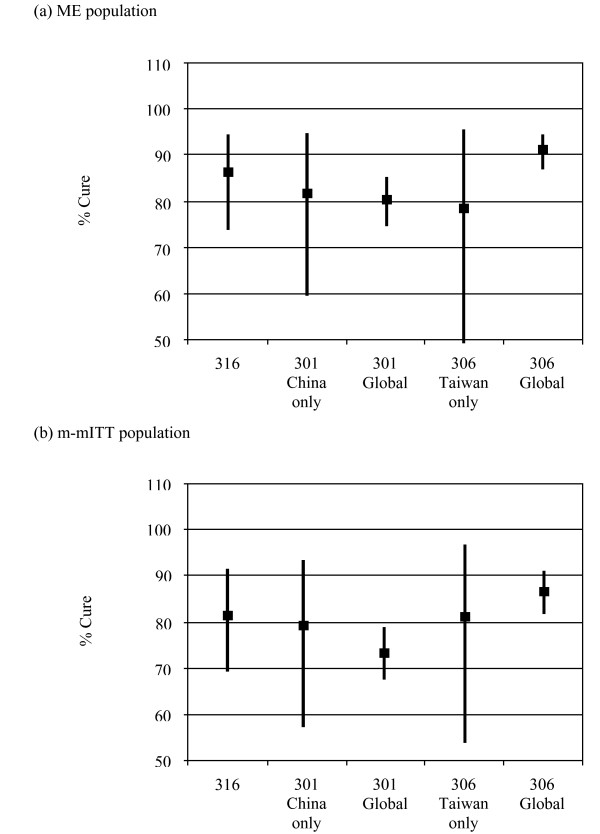
**Tigecycline clinical cure rates compared to previous intra-abdominal studies (301, 306): (a) microbiologically evaluable (ME) and (b) microbiologic modified intent-to-treat (m-mITT) populations**. Error bars indicate 95% confidence intervals, unweighted and calculated using the method of Clopper and Pearson.

These findings in Chinese patients were similar to results in 2 global double-blind clinical trials [16], wherein the clinical response of tigecycline was found to be non-inferior to imipenem/cilastatin (Figure 2). The results of the Breslow-Day test indicate that there was no significant difference in clinical response across the 3 studies in the ME (*P *= 0.0979) or the m-mITT population (*P *= 0.1655) for the current study in Chinese patients (Study 316) and the 2 pivotal global studies (Study 301 and Study 306).

A total of 6 tigecycline- and 3 imipenem/cilastatin-treated patients in the ME population had positive pretherapy blood culture results, including 7 isolates in tigecycline patients and 4 in imipenem/cilastatin patients. All blood culture isolates were Gram-negative rods. All 9 patients with bacteremia were reported as having a clinical cure/bacteriologic eradication at the TOC visit.

### Microbiologic efficacy

For the ME population, presumed eradication of intra-abdominal isolates at the patient level based upon clinical response mirrored the clinical cure rates: 86.5% for tigecycline- and 97.9% for imipenem/cilastatin-treated patients (Table 5). Eradication rates stratified by monomicrobial and polymicrobial infection are also summarized in Table 5.

**Table 5:** Microbiologic response by patient at test-of-cure visit (ME population)

	Tigecycline		Imipenem/cilastatin		Difference (Tigecycline-Imipenem/cilastatin)
	**N**	**% (95% CI)**	**N**	**% (95% CI)**	**% (95% CI)**

**Overall**					
Eradication	45/52	86.5 (74.2, 94.4)	47/48	97.9 (88.9, 99.9)	-11.4 (-23.5, 0.7)
Persistence	7/52	13.5 (5.6, 25.8)	1/48	2.1 (0.1, 11.1)	
Superinfection	0/52	0 (0.0, 6.8)	0/48	0 (0.0, 7.4)	
**Monomicrobial**					
Eradication	30/33	90.9 (75.7, 98.1)	25/26	96.2 (80.4, 99.9)	-5.2 (-22.0, 13.7)
Persistence	3/33	9.1 (1.9, 24.3)	1/26	3.8 (0.1, 19.6)	
Superinfection	0/33	0 (0.0, 10.6)	0/26	0 (0.0, 13.2)	
**Polymicrobial**					
Eradication	15/19	78.9 (54.4, 93.9)	22/22	100.0 (84.6, 100.0)	-21.1 (-46.1, 2.2)
Persistence	4/19	21.1 (6.1, 45.6)	0/22	0 (0.0, 15.4)	
Superinfection	0/19	0 (0.0, 17.6)	0/22	0 (0.0, 15.4)	

Eradication rates at the TOC visit for the most common types of isolated intra-abdominal pathogens are outlined in Table 6 for the two treatment groups. For *E. coli*, the most commonly isolated bacteria, eradication rates were 88.1% for tigecycline vs. 97.7% for imipenem/cilastatin. There were no obvious differences in eradication rates of other aerobic and anaerobic bacteria, although the number of isolates per species was small, making comparisons difficult.

**Table 6:** Microbiologic eradication at the isolate level: selected baseline isolates at test-of-cure visit (ME population)

	Tigecycline (N = 52)			Imipenem/cilastatin (N = 48)		
**Baseline Isolate**	**N**	**E**	**ER (%)**	**N**	**E**	**ER (%)**

**Total**	83	69	83.1	75	74	98.7
Gram negative aerobic bacteria	56	48	85.7	59	58	98.3
*E. coli*	42	37	88.1	44	43	97.7
Other Enterobacteriaceae	8	6	75.0	9	9	100
Non-fermentative Gram-negative bacilli	6	5	83.3	6	6	100
Gram positive aerobic bacteria	15	12	80.0	8	8	100
Enterococcus spp.	10	8	80.0	5	5	100
Streptococcus spp.	5	4	80.0	2	2	100
Staphylococcus spp.	0	0	0	1	1	100
Anaerobe	12	9	75.0	8	8	100
Bacterioides spp.	7	5	71.4	6	6	100
Other anaerobe	5	4	80.0	2	2	100

E = eradication (presumed), and ER = eradication rate.

Overall, MIC values for tigecycline against the most commonly isolated aerobes and anaerobes was ≤2.0 μg/mL. For *E. coli *(n = 86), MIC_50 _and MIC_90 _values were 0.125 μg/mL and 0.5 μg/mL for tigecycline, respectively, and 0.25 μg/mL and 0.5 μg/mL for imipenem/cilastatin, respectively. For patients in the tigecycline group who had persistent *E. coli *infections (5 isolates; 11.9%), MIC values ranged from 0.125 to 0.5 μg/mL. Bacterial susceptibilities to tigecycline were consistent with clinical responses, and no isolates from later cultures with a decreased susceptibility (≥4-fold increase in MIC from baseline) to tigecycline were identified.

### Safety and tolerability

The mITT population received a median of 5 and 6 days of tigecycline or imipenem/cilastatin treatment, respectively. The overall range for the number of days of therapy was 2 to 14 days. The overall incidence of treatment-emergent AEs was 80.4% for tigecycline vs. 53.9% for imipenem/cilastatin therapy (*P *< 0.001); this difference between treatment groups was primarily due to gastrointestinal-related events, primarily nausea and vomiting (Table 7). The overall percentage of subjects with study drug-related AEs was significantly higher in the tigecycline group compared to the imipenem/cilastatin treatment arm (55.7% vs. 41.2%; *P *< 0.05). The difference stemmed primarily from between-group differences in nausea (20.6% tigecycline vs. 2% imipenem/cilastatin; *P *< 0.001) and vomiting (tigecycline 10.3% vs. imipenem/cilastatin 1%; *P *= 0.004). Most AEs were mild to moderate in severity and not considered clinically important.

**Table 7:** Common treatment-emergent adverse events (≥3% in either group)

Body System Adverse Event	Tigecycline (N = 97)	Imipenem/cilastatin (N = 102)	*P *value^a^
Any adverse event	78 (80.4)	55 (53.9)	< 0.001
Body as a whole	11 (11.3)	8 (7.8)	0.473
Chest pain	3 (3.1)	1 (1.0)	0.359
Digestive system	35 (36.1)	16 (15.7)	0.001
Abdominal distension	7 (7.2)	2 (2.0)	0.094
Diarrhea	5 (5.2)	9 (8.8)	0.409
Nausea	21 (21.6)	4 (3.9)	< 0.001
Vomiting	12 (12.4)	2 (2.0)	0.005
Hemic and lymphatic system	23 (23.7)	21 (20.6)	0.613
Anemia	3 (3.1)	3 (2.9)	1.000
Coagulation disorder	4 (4.1)	0 (0)	0.055
Monocytosis	1 (1.0)	6 (5.9)	0.119
Prothrombin time prolonged	1 (1.0)	4 (3.9)	0.369
Thrombocythemia	6 (6.2)	6 (5.9)	1.000
Thrombocytopenia	5 (5.2)	1 (1.0)	0.111
Metabolic and nutritional	37 (38.1)	35 (34.3)	0.658
ALT/SGPT increased	3 (3.1)	5 (4.9)	0.722
AST/SGOT increased	6 (6.2)	7 (6.9)	1.000
Amylase increased	8 (8.2)	9 (8.8)	1.000
Bilirubinemia	21 (21.6)	12 (11.8)	0.085
Healing abnormal	7 (7.2)	6 (5.9)	0.779
Hyperglycemia	1 (1.0)	4 (3.9)	0.369
Hypocalcemia	6 (6.2)	5 (4.9)	0.763
Hypokalemia	1 (1.)	4 (3.9)	0.369
Hyponatremia	3 (3.1)	1 (1.0)	0.359
Hypophosphatemia	5 (5.2)	5 (4.9)	1.000
Hypoproteinemia	10 (10.3)	11 (10.8)	1.000
Lipase increased	3 (3.1)	0 (0)	0.114
Respiratory system	3 (3.1)	3 (2.9)	1.000
Cough increased	3 (3.1)	1 (1.0)	0.359
Skin and appendages	1 (1.0)	4 (3.9)	0.369
Rash	1 (1.0)	4 (3.9)	0.369
Adverse event associated with miscellaneous factors	4 (4.1)	6 (5.9)	0.748
Local reaction to procedure	4 (4.1)	6 (5.9)	0.748

AST/SGOT = aspartate aminotransferase/serum glutamic oxaloacetic transaminase.

ALT/SGOT = alanine aminotransferase/serum glutamic oxaloacetic transaminase.

^a^Between-group comparisons of adverse events were analyzed by using the Fisher exact test. Significant between-group difference at *P *≤ 0.05 level.

Nine SAEs were recorded during the study period (8 [8.2%] tigecycline vs. 1 [1.0%] imipenem/cilastatin) (*P *= 0.016). The most frequently reported SAE was abnormal healing (3 [3.1%] tigecycline vs. 0 imipenem/cilastatin; *P *= 0.114). There were no significant differences between the treatment groups in the specific types of SAEs experienced. Overall, these SAEs were not considered by the investigator to be related to study medication.

Two (2.1%) tigecycline- and 2 (2.0%) imipenem/cilastatin-treated patients had treatment stopped early because of an adverse event. In the tigecycline group, 1 patient discontinued therapy because of gastrointestinal hemorrhage and shock and 1 because of nausea. Both imipenem/cilastatin-treated patients discontinued treatment secondary to diarrhea.

A single death was reported during the study, which was unrelated to study medication. This tigecycline-treated patient, a 74-year-old man who underwent a colostomy, died of septic shock and multiple organ failure 1 day after his first dose of study medication.

The only clinically relevant laboratory test abnormality was that significantly more Chinese patients treated with tigecycline had a low platelet count (i.e., platelet count ≤ 100 × 10^9^/L) compared with the imipenem/cilastatin-treated patients (11.5% vs. 2.0%, *P *= 0.009). Further examination of 7 patients who had platelet counts ≤ 50 × 10^9^/L revealed that these patients either had thrombocytopenia at baseline, or thrombocytopenia was attributed by the investigator to infection/sepsis. Mean platelet count increases observed at the TOC assessment were similar in both treatment arms. There were no significant differences between the treatment groups with respect to mean changes in individual ECG parameters, nor were there any AEs associated with prolonged QT interval after either treatment reported.

## Discussion

This analysis of hospitalized Chinese patients demonstrated that open-label tigecycline monotherapy (100 mg initial dose, followed by 50 mg q12 hours) was effective for the treatment of cIAIs. The 199 patients included in the mITT population had mild to moderately severe intra-abdominal infections, as described by a mean APACHE II score of 4.6. For patients comprising the ME and m-mITT populations, clinical cure rates ranged from 81.7% to 86.5% for tigecycline vs. 90.9% to 97.9% for imipenem/cilastatin at the test-of-cure visit. All ME patients with bacteremia in both treatment groups were clinically cured; however, the absolute number of such patients was small. Tigecycline-treated patients with polymicrobial infection tended to have lower clinical cure rates compared with patients who had monomicrobial infection. Although approximately three-quarters of patients had complicated appendicitis, tigecycline was effective across the range of clinical diagnoses.

Microbiologic responses paralleled clinical outcomes for both tigecycline and imipenem/cilastatin in Chinese patients. In the ME population, the baseline organisms were eradicated in 86.5% of tigecycline-treated patients and 97.9% of patients treated with imipenem/cilastatin at the TOC visit. Satisfactory eradication of commonly encountered aerobic and anaerobic intestinal bacteria after both treatments was also observed. Eradication rates for *E. coli*, the most commonly isolated bacteria, were 88.1% for tigecycline vs. 97.7% for imipenem/cilastatin. Although other Gram-negative enterics (e.g., *K. pneumoniae, P. mirabilis*), Gram-positive (e.g., *Streptococcus *spp., *Enterococcus *spp.), and anaerobic bacteria (e.g., *B. fragilis*, *Bacteroides *spp.) were isolated in small numbers, tigecycline generally eradicated most of these pathogens. Overall, these data provide in vivo evidence that tigecycline has broad-spectrum activity against common bacterial etiologies associated with cIAIs [9,10,14,17,18].

Tigecycline monotherapy was generally well tolerated in this study population, even though the overall incidence of treatment-emergent AEs was significantly higher after tigecycline therapy (80.4%) compared with imipenem/cilastatin (53.9%; *P *< 0.001). The main reason for the increased rates of AEs was gastrointestinal, with tigecycline-treated patients having a two-fold higher rate than those given imipenem/cilastatin (*P *= 0.001). In particular, nausea and vomiting occurred significantly more often (5-6 fold) after tigecycline therapy. To the contrary, diarrhea occurred in higher rates among imipenem/cilastatin recipients (*P *> 0.05). Gastrointestinal-related adverse events rarely led to early discontinuation of either therapy, however, as only one tigecycline patient stopped treatment early due to nausea, as did two imipenem/cilastatin-treated patients secondary to diarrhea. Of interest, nearly two-fold more patients treated with tigecycline had bilirubinemia compared with patients treated with imipenem/cilastatin (21.6% vs. 11.8%; *P *= 0.085). While bilirubinemia has been observed in prior studies of tigecycline to treat cIAI, the rates in the current study were higher than those observed in patients enrolled in previous trials (16); in each instance, however, the presence of mitigating factors confound determinations of a relationship between the study drugs and changes in bilirubin levels. Overall, safety/tolerability findings described herein were consistent with the original global trials [16] and support previous safety data from phase 2 and 3 studies [16,19-24]. Significantly more subjects in the tigecycline treatment arm had SAEs than subjects in the imipenem/cilastatin arm (8.2% vs. 1.0%, *P *= 0.016); however, there were no significant differences between the treatment groups in the specific type of SAE reported. The most frequently reported SAE was abnormal healing. No unusual or novel adverse events were reported in Chinese patients after tigecycline monotherapy.

A major limitation of the current trial is that it was not powered for a formal statistical analysis of noninferiority. In addition, the study design was open-label (unblinded), adding to the confounding factors affecting comparisons of efficacy within the study. Accordingly, it is inappropriate to draw conclusions about the efficacy of tigecycline on the basis of the results of this study alone. However, the results of our study in Chinese patients is consistent with the efficacy findings from 2 global randomized, double-blind studies [16], and the same dose administration schedule was used across the 3 studies to treat hospitalized patients with cIAI. In study 301, 80.6% of tigecycline-treated patients and 82.4% of imipenem/cilastatin-treated patients in the ME population were clinically cured, as were 73.5% of tigecycline-treated patients and 78.2% of imipenem/cilastatin-treated patients in the m-mITT population. In the overall ME population of study 306, 91.3% of tigecycline-treated patients and 89.9% of imipenem/cilastatin-treated patients were clinically cured; in the m-mITT population, 86.6% of tigecycline-treated subjects and 84.6% of imipenem/cilastatin-treated subjects were clinically cured. Furthermore, the 2 global studies included sites that enrolled Chinese patients [16]. Formal statistical analyses in the global studies demonstrated that tigecycline met the statistical criteria of non-inferiority to imipenem/cilastatin for the primary endpoint of clinical response (cure or failure) in the co-primary populations at the TOC assessment, and the results of primary clinical cure or failure analyses for tigecycline in the study described herein mirrored the results in these pivotal cIAI studies.

## Conclusion

In summary, tigecycline monotherapy appears to be both effective and safe for the treatment of cIAI in Chinese patients. Both the efficacy and safety analyses for this study are consistent with the profile of tigecycline elucidated in the pivotal trials (Study 301/306) [16]. Digestive-related AEs were significantly higher in the tigecycline group, especially nausea and vomiting. With the diverse bacteriology of cIAIs and the emergence of bacterial resistance, tigecycline provides an empiric monotherapy option with coverage against a broad range of Gram-positive and Gram-negative aerobic and anaerobic bacteria, including resistant isolates based upon in vitro data.

## Competing interests

This study and analysis was sponsored by Wyeth Research, Collegeville, PA, USA, which was acquired by Pfizer, Inc. in October 2009. Pfizer assumes responsibility for the study design, and collection, analysis, and interpretation of the data. Pfizer provided the authors with editorial support for the preparation of this publication (Upside Endeavors, LLC, Sanatoga, PA, USA).

Zhangjing Chen, Robert Maroko, and Angel Cooper are employees of Pfizer, Inc. Zhangjing Chen was one of the investigators in studies 316 and 301.

Jufang Wu, Yingyuan Zhang, Junming Wei, Xisheng Leng, Jianwei Bi, Rong Li, Lunan Yan, Zhiwei Quan, Xiaoping Chen, Yunsong Yu, Zhiyong Wu, Dawei Liu, and Xiaochun Ma have no competing interests to declare.

## Authors' contributions

ZC, J Wu, YZ, J Wei, XL, JB, RL, LY, ZQ, XC, YY, XW, DL, and XM conducted the study, contributed to data acquisition, and reviewed and approved the draft manuscript. RM and AC contributed to, reviewed, and approved the draft manuscript. All authors reviewed and approved the final version.

## Pre-publication history

The pre-publication history for this paper can be accessed here:

http://www.biomedcentral.com/1471-2334/10/217/prepub
